# Tricarboxylic Acid Cycle Metabolites as Mediators of DNA Methylation Reprogramming in Bovine Preimplantation Embryos

**DOI:** 10.3390/ijms21186868

**Published:** 2020-09-18

**Authors:** Jessica Ispada, Aldcejam Martins da Fonseca Junior, Camila Bruna de Lima, Erika Cristina dos Santos, Patricia Kubo Fontes, Marcelo Fábio Gouveia Nogueira, Vinicius Lourenço da Silva, Fernanda Nascimento Almeida, Saul de Castro Leite, James Lee Chitwood, Pablo Juan Ross, Marcella Pecora Milazzotto

**Affiliations:** 1 Laboratory of Embryonic Metabolism and Epigenetics, Center of Natural and Human Sciences, Federal University of ABC, Santo André, 09210-580 São Paulo, Brazil; jessica.ispada@usp.br (J.I.); aldcejamjunior@gmail.com (A.M.d.F.J.); lima.camilab@gmail.com (C.B.d.L.); erika.cristina@ufabc.edu.br (E.C.d.S.); 2 Institute of Biomedical Sciences, University of Sao Paulo, 05508-000 São Paulo, Brazil; 3 Centre de Recherche en Reproduction, Développement et Santé Intergénérationnelle (CRDSI), Département des Sciences Animales, Faculté des Sciences de l’Agriculture et de l’Alimentation, Université Laval, Québec, QC G1V 0A6, Canada; 4 Laboratory of Phytomedicines, Pharmacology and Biotechnology, Department of Pharmacology, Institute of Biosciences, São Paulo State University (Unesp), Campus of Botucatu, 18618-689 São Paulo, Brazil; pkfontes@gmail.com (P.K.F.); marcelo.fabio@unesp.br (M.F.G.N.); 5 Department of Biological Sciences, School of Sciences and Languages, São Paulo State University (Unesp), Campus of Assis, 19806-900 São Paulo, Brazil; 6 Bioinformatics and Health Informatics Group, Center for Engineering, Modeling and Applied Social Sciences, Universidade Federal do ABC, São Bernardo do Campo, 09606-045 São Paulo, Brazil; vinicius.lou.silva@gmail.com (V.L.d.S.); fernanda.almeida@ufabc.edu.br (F.N.A.); 7 Center for Mathematics Computation and Cognition, Universidade Federal do ABC, Santo André, 09210-580 São Paulo, Brazil; saul.leite@ufabc.edu.br; 8 Department of Animal Science, University of California Davis, Davis, CA 95616, USA; jlchitwood@ucdavis.edu (J.L.C.); pross@ucdavis.edu (P.J.R.)

**Keywords:** bovine, embryo, metabolism, epigenetic, DNA methylation

## Abstract

In many cell types, epigenetic changes are partially regulated by the availability of metabolites involved in the activity of chromatin-modifying enzymes. Even so, the association between metabolism and the typical epigenetic reprogramming that occurs during preimplantation embryo development remains poorly understood. In this work, we explore the link between energy metabolism, more specifically the tricarboxylic acid cycle (TCA), and epigenetic regulation in bovine preimplantation embryos. Using a morphokinetics model of embryonic development (fast- and slow-developing embryos), we show that DNA methylation (5mC) and hydroxymethylation (5hmC) are dynamically regulated and altered by the speed of the first cleavages. More specifically, slow-developing embryos fail to perform the typical reprogramming that is necessary to ensure the generation of blastocysts with higher ability to establish specific cell lineages. Transcriptome analysis revealed that such differences were mainly associated with enzymes involved in the TCA cycle rather than specific writers/erasers of DNA methylation marks. This relationship was later confirmed by disturbing the embryonic metabolism through changes in α-ketoglutarate or succinate availability in culture media. This was sufficient to interfere with the DNA methylation dynamics despite the fact that blastocyst rates and total cell number were not quite affected. These results provide the first evidence of a relationship between epigenetic reprogramming and energy metabolism in bovine embryos. Likewise, levels of metabolites in culture media may be crucial for precise epigenetic reprogramming, with possible further consequences in the molecular control and differentiation of cells.

## 1. Introduction

Epigenetic reprogramming is essential for embryonic development and includes large changes in DNA methylation and histone modifications. DNA methylation reprogramming following fertilization includes active cytosine demethylation of the paternal genome promoted by TET enzymes (ten-eleven translocation), while the maternal genome undergoes mostly passive demethylation, except for imprinted genes and some repetitive elements that remain methylated throughout development. De novo methylation initiates around the 8–16 cells stage and reaches somatic cell levels by the late blastocyst stage for in vitro-produced bovine embryos [1].

Changes in the epigenetic profile are required for proper embryonic development and may be affected by environmental factors. Particularly during the early stages of embryonic development, cells need to make a number of decisions that are crucial for embryo survival. Each choice—proliferation, differentiation or death—is made in a dynamic environment that is subjected to modifications, from slight changes to stressful challenges. Thus, it is unlikely that the epigenome and, consequently, the molecular control of blastomeres’ fate would be refractory to metabolic changes induced in response to the environment surrounding the embryo. Indeed, in other cell types, the activity of several enzymes responsible for inserting or removing epigenetic modifications are regulated, at least in part, by the presence and amount of energy metabolism substrates or cofactors that cross the pores of the nuclear envelope, paving the way for information exchange between metabolism and the molecular control of transcription [2,3].

The crosstalk between epigenetic reprogramming and metabolism was defined as metaboloepigenetics [4] and is better described for embryonic stem cells. One example of how metabolism and epigenetics are interconnected in these cells is how a high intracellular α-ketoglutarate (α-KG)/succinate ratio allows the maintenance of high TET activity and reduced DNA methylation, whereas cells at more differentiated states, such as somatic cells, present a lower α-KG/succinate ratio and consequently lower TET activity and higher DNA methylation levels [2]. Similarly, a recent study demonstrated that α-KG levels affect Tet and Dnmt3 activity, increasing the ratio of 5-hydroxymethylcytosine/5-methylcytosine (5hmC/5mC) in murine embryos [5].

During initial cleavage divisions, bovine embryos use pyruvate as the main substrate for energy production, possibly in response to the lower energy demands at this stage [6]. After the time of embryonic genome activation (at the 8–16 cell stage), the demand for ATP increases as the embryo requires more energy to support the intense cell proliferation, blastocoel formation and expansion. Thus, there is an increment in glucose consumption, glycolytic pathway activity as well as the tricarboxylic acid (TCA) cycle and oxidative phosphorylation, with consequences for the intracellular balance of intermediate metabolites [7,8]. As embryos with dissimilar dynamics during the first cell cycles present remarkable differences in molecular and metabolic characteristics, we hypothesized that these differences could influence their epigenetic reprogramming ability [9,10,11,12].

The timing to complete the first cell divisions, in particular from 2 to 5 cells, is a well-studied predictor of blastocyst formation and implantation in humans [13,14]. In the bovine, the kinetics of the first two cell divisions are associated with distinct phenotypes regarding blastocyst conversion, energy and lipid metabolism, stress response and transcriptomic and methylome profile at the blastocyst stage. Despite that, other parameters such as sex ratio, total cell number and specially morphology in embryos from day 7 of culture remain unaltered [10,11]. Further, the metabolic and epigenetic differences observed between these groups of embryos are preserved when sexed embryos are produced using this kinetics model [12].

Here, we describe the dynamics of DNA methylation (5mC) and hydroxymethylation (5hmC) of embryos with different developmental kinetics. Transcriptome analysis revealed that specific writers and erasers of these DNA modifications were barely affected, while TCA cycle genes were mostly upregulated in slow-developing blastocysts, suggesting that energy metabolism could be driving epigenetic changes by modulating enzymes activity. We confirmed this link between metabolism and epigenetics by modulating intermediate metabolites of the TCA cycle in culture media, which led to changes in DNA methylation in a dose-dependent manner. We finally discuss the importance of these modifications during the first stages of mammalian development and how in vitro culture systems should be carefully refined to prevent epigenetic abnormalities induced by metabolism.

## 2. Results

### 2.1. DNA Methylation Reprogramming Failure in Slow Developing Embryos

**Figure 1:**
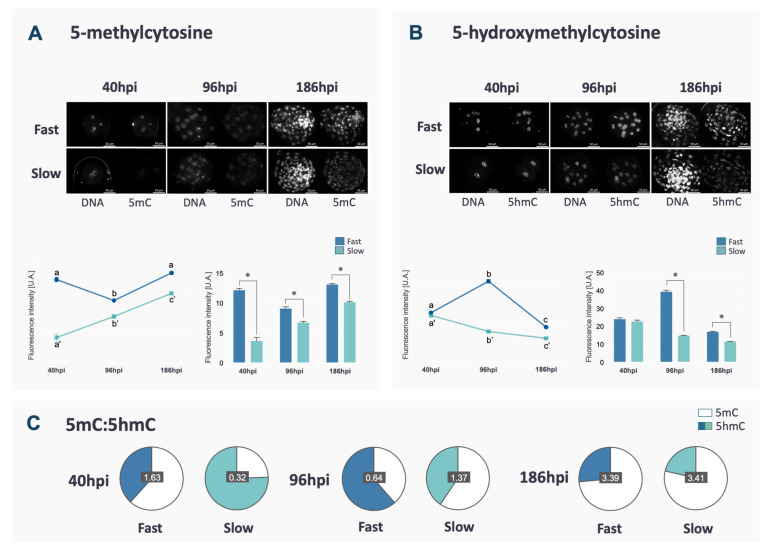
Fluorescence intensity and representative images (original magnification 400×) of 5mC (**A**) and 5hmC (**B**) of fast and slow embryos at 40, 96 and 168 hpi. Nuclei were stained with propidium iodate and immunostained with antibodies against 5mC or 5hmC. Fluorescence levels for each mark are represented. Differences among time points within the same group are indicated by lower case letters (a, b, c) and those between fast and slow embryos for each time point are indicated with *. Data are represented as mean ± S.E.M. The ratio between 5mC and 5hmC for each group in each time point is represented in (**C**) to demonstrate the overall change in proportion between the two marks along the development, although the proportion of 5mC and 5hmC was not statistically compared.

DNA methylation reprogramming follows a specific and well-established pattern in bovine embryos. In this study, 5mC and 5hmC were assessed in fast and slow embryos to verify if the kinetics of the first cleavages could be associated with failures in DNA methylation reprogramming. Fast embryos showed a characteristic reduction between 40 and 96 hpi, followed by an increase at the blastocyst stage (*p* < 0.0001, Figure 1A), matching the profile of DNA demethylation/methylation often described in the literature. Differently, in slow embryos, the levels of 5mC increased throughout the development (*p* < 0.0001, Figure 1A).

The profile of 5hmC, a step in the DNA demethylation process, was also evaluated and, in both groups, it was the opposite of 5mC (*p* < 0.0001, Figure 1B). It is important to point out that slow embryos had lower 5mC and 5hmC levels compared to fast embryos at all analyzed time points (*p* < 0.0001, Figure 1A,B) except for 5hmC at 40 hpi (*p* > 0.1). At 40 hpi, slow embryos also presented a lower ratio of 5mC/5hmC, while at 96 and 186 hpi, a lower ratio of 5mC/5hmC was observed in fast embryos (Figure 1C). At the blastocyst stage, total cell number was similar between fast and slow (Supplementary Figure S1A). Besides, the fluorescence intensity of DNA marks (5mC and 5hmC) varies between ICM and TE in both kinetics groups, as demonstrated in Supplementary Figure S1B.

**Figure 2:**
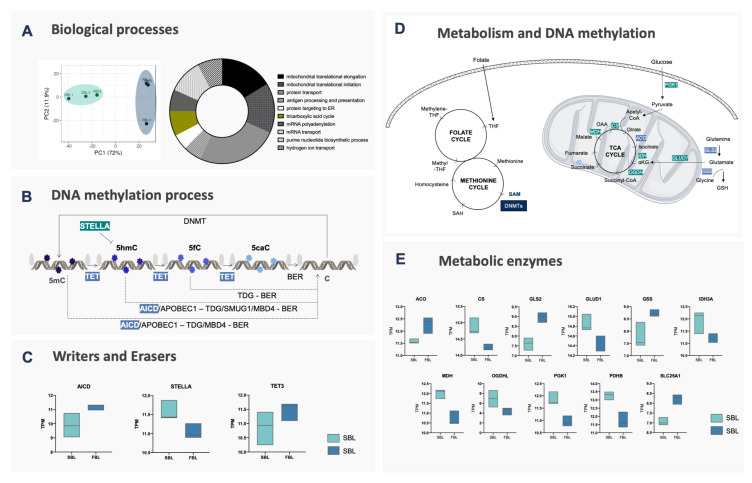
Molecular evidence for fast and slow blastocysts. (**A**) PCA and gene ontology analysis of RNA-Seq data. Fast and slow blastocysts are highlighted in light blue and light green, respectively. The most affected biological processes are indicated in the Doughnut chart, according to the number of genes involved in each pathway. Genes from the TCA cycle are highlighted in olive green; (**B**) enzymes that act on DNA methylation and demethylation processes. DNA cytosines (cytosines—C) are methylated by DNMT1, DNMT3A or DNMT3B enzymes, generating 5-methylcytosines (5mC), by transferring the methyl group from S-adenosylmethionine (SAM) donors. 5mC can be protected from demethylation by STELLA or, with the use of α-ketoglutarate (α-KG), be oxidized by TET1-3 enzymes to 5-hydroxymethylcytosine (5hmC), 5-carboxylcytosine (5caC) or 5-formylcytosine (5fC). These last two can be glycosylated by TDG and follow to the base excision repair (BER—composed of: NEIL3, NEIL2, LIG3, XRCC1 and APEX1) mechanism, resulting in the replacement for an unmethylated cytosine. Besides, 5mC and 5hmC can be converted by AICDA or APOBEC1 to Thymine (T) and 5-methyluracil (5hmU), respectively, the first being removed by TDG/MBD4 and the second by TDG/SMUG1; (**C**) boxplot of genes related to the DNA methylation process identified as differentially expressed between fast (light blue) and slow (light green) blastocysts; (**D**) enzymes involved in the metabolic process related to the DNA methylation process; (**E**) boxplot of genes related to metabolic pathways identified as differentially expressed between fast (light blue) and slow (light green) blastocysts. Genes related to the one-carbon cycle (1C) were similar between groups, however, enzymes involved in glycolysis (*PGK1*), TCA cycle progression (*CS*, *IDH3A*, *MDH* and *OGDHL*) and glutamate conversion to α-KG (*GLUD1*) were upregulated in slow embryos. Citrate conversion to isocitrate (*ACO*) and citrate export from mitochondria (*SLC25A1*) were upregulated in FBL. *GLS* and *GSS*, both related to glutamine conversion to glutamate and glutathione, were also upregulated in FBL.

To better understand the regulation of DNA methylation and demethylation in fast and slow embryos, previously reported RNA-Seq results were revisited [10]. Principal component analysis (PCA) revealed a clear distinction between fast blastocysts (FBL) and slow blastocysts (SBL) groups (Figure 2A). Therefore, we assessed the levels of transcripts involved in DNA methylation reprogramming (Figure 2B,C, and Supplementary Table S1). Significant differences were only observed for developmental pluripotency associated 3 (*STELLA*) that was upregulated, and *TET3* and amyloid precursor protein intracellular domain (*AICD*) that were downregulated in SBL embryos, suggesting an inability of SBL to promote DNA demethylation efficiently.

### 2.2. Metabolic Modulation Is Involved with Epigenetic Reprogramming

In stem cells, it has been shown that energy metabolism can directly and indirectly modulate epigenetic marks, either by acting as methyl donors (methyl from S-adenosyl methionine) or providing cofactors for activating/deactivating specific enzymes (α-KG and succinate influencing TET activity) [2,15]. Interestingly, in the present study, differentially expressed genes obtained by RNA-Seq were submitted to gene ontology analysis, which indicated the TCA cycle as one of the ten most affected biological processes between fast and slow embryos. Added to the fact that the gene expression of enzymes involved in DNA methylation/demethylation only partially explained the different patterns of epigenetic reprogramming observed between fast and slow blastocysts, we hypothesized that energy metabolism enzymes could also play a role in the modulation of epigenetic mechanisms during embryonic development. To confirm this hypothesis, first we selected genes related to these processes and then assessed their profile in FBL and SBL (Supplementary Table S1). As a result, several genes from the TCA cycle were found to be upregulated in the SBL blastocysts. These genes included *PGK1* (involved in glycolysis), Pyruvate Dehydrogenase E1 Subunit Alpha 1 (*PDK1*), Malate dehydrogenase (*MDH*), glutamate dehydrogenase (*GLUD1*), citrate synthase (*CS*), Isocitrate dehydrogenase (*IDH3b*) and α-ketoglutarase dehydrogenase (*OGDHL*), suggesting alterations to the a-KG/succinate ratio (Figure 2D,E).

**Figure 3:**
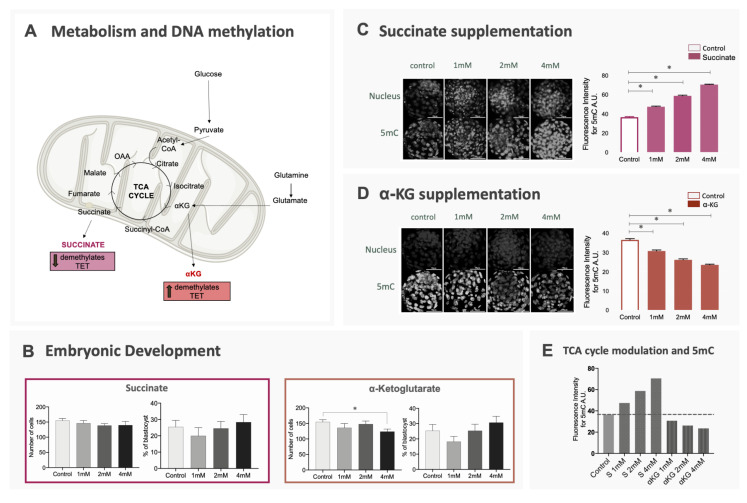
Effect of changes in the availability of α-KG and succinate on embryo development and DNA methylation. Bovine embryos were produced in vitro and culture in media supplemented with different doses of α-ketoglutarate, succinate or without supplementation (control). (**A**) TCA cycle and the relation between its metabolites and the activity of enzymes involved in DNA methylation; (**B**) blastocyst rates and total cell number of blastocysts for all groups. Data are represented as mean ± S.E.M; (**C**,**D**) 5mC immunostaining of blastocysts cultured in succinate or α-KG. Data are represented as mean ± S.E.M. For all comparisons described above, * represents statistical differences; (**E**) representation of mean methylation levels in blastocysts for each experimental group compared to control.

The next step was to confirm the relationship between energy metabolism and the regulation of methylation/demethylation processes. For the purpose, we modulated the availability of α-KG and succinate, both substrates of the TCA cycle, during embryo culture, expecting that higher levels of α-KG would increase the activity of TET enzymes (promoting DNA demethylation), while higher levels of succinate could reduce the activity of these enzymes (Figure 3A) [2]. This modulation was performed with the use of dimethyl-α-KG and dimethyl-succinate, both analogues that are membrane-permeable and were already reported to exert a modulatory effect in the TCA cycle [2]. As expected, embryos cultured with α-KG supplementation presented lower levels of 5mC at the blastocyst stage, while blastocysts cultured with succinate supplementation had increased levels of 5mC (*p* < 0.0001) (Figure 3C–E). These differences also occurred when TE and ICM cells were evaluated separately for 5mC fluorescence intensity (Supplementary Figure S1C,D). Interestingly, despite the differences in DNA methylation, blastocyst rates did not differ when the groups were compared to controls and only blastocysts cultured in 4 mM of α-KG presented a difference in total cell number (*p* = 0.013) (Figure 3B). Supplementation with α-KG or succinate was capable of altering global DNA methylation levels, confirming that energy metabolism is an important regulator for this epigenetic mark in bovine embryos.

## 3. Discussion

Molecular and metabolic adaptations occurring during the preimplantation phase are determinant to the development of viable embryos and healthy offspring. In this context, molecular aspects such as global gene expression and epigenetic reprogramming are responsible for activating proper biological pathways to ensure embryo survival and continued development. Metabolic aspects are related to the activation of specific pathways to adjust the energy supply needed at each stage of development.

Recent studies demonstrated that the kinetics model is very valuable in the study of molecular and metabolic pathways [10]. This is due to the fact that fast and slow embryos present consistent differences in terms of gene expression, stress response and energy production mechanisms [10,11], which is particularly intriguing as they are morphologically similar. These molecular and metabolic observations led us to hypothesize that the said differences could be a consequence of the constant crosstalk between energy metabolism and epigenetic reprogramming, i.e., metabolites modulate epigenetics through energy metabolism enzymes, but epigenetic mechanisms can also impact the metabolic profile [2,5,16,17].

The demethylation/de novo methylation cycle is expected during normal preimplantation development to remove parental marks, except for imprinted genes and some repetitive elements that remain methylated, and is essential to support the development of a new set of unspecialized pluripotent cells [1,18,19,20,21]. In this work, we observed that in fast embryos, the demethylation/de novo methylation pattern is more similar to that described in the literature for in vivo and in vitro bovine embryos [22,23,24]. Slow embryos, on the other hand, presented a progressive increase in DNA methylation due to the first cleavages and a decrease in 5hmC throughout development, demonstrating the inability of these embryos to correctly promote DNA demethylation between the two-cell and blastocyst stages.

Based on data obtained at 40 hpi, we speculated that the difference in DNA methylation levels could be reflecting the difference in the number of cells (fast embryos with four cells vs. slow embryos with two or three cells). In this scenario, fast embryos should have undergone more cell cycles and consequently more passive demethylation steps yielding lower DNA methylation levels. Interestingly, the very opposite phenomenon was observed, and fast embryos had increased 5mC compared to slow embryos at all time points. Particularly at the blastocyst stage, the higher levels of 5mC in fast embryos corroborate our previous methylome analysis, in which fast embryos presented 7976 hypermethylated regions distributed throughout the entire genome against only 3608 hypermethylated regions in slow embryos, mostly in cytosine-guanine (CpG) islands [12]. Furthermore, in agreement with previous reports [11], there was no difference in the number of cells between FBL and SBL, demonstrating that the number of cells equates during development. 

At the blastocyst stage, slow embryos had increased levels of *STELLA*, known to protect DNA methylation and prevent demethylation [21], and lower levels of *TET3* and *AICD*, both involved in the DNA demethylation process. *TET3* is the *TET* responsible for, at the earliest stages of development, promoting active demethylation of the paternal genome [25]. *TET3*-deficient embryos fail to demethylate the paternal *OCT4* and *NANOG* genes, delaying the activation of *OCT4* and impairing cell reprogramming [26]. Besides, it is known that the typical embryonic reprogramming includes an important DNA demethylation step, thus the expression profile of these genes might be the remaining response of slow embryos to prevent the signaling for demethylation, likely an attempt to preserve DNA methylation marks that were scarce in this group since the earliest developmental stages. Contrarily, fast blastocysts present more 5mC and seem to coordinate the DNA methylation status in a more organized manner.

Raising the possibility that modulators of DNA methylation remodeling are metabolites, we assessed the transcriptional pattern of DNA methyltransferases that use the S-adenosylmethionine (SAM) generated in the one-carbon cycle (1C) as the substrate for DNA methylation (reviewed by [27]). However, we did not observe differential transcription of these enzymes, once again suggesting that differences in DNA methylation levels could be a result of failure in promoting demethylation, rather than the addition of methylations.

Interestingly, slow embryos presented higher transcription of enzymes involved in glycolysis and the TCA cycle, such as *PGK1*, *PDHB*, *IDH*, *OGDH*, *MDH* and *CS.* We speculate that the higher activity of TCA most likely indicates that the majority of α-KG is being redirected to energy generation rather than other intracellular functions. The reduction in α-KG availability ultimately impacts the regulation of TET activity, explaining why slow embryos are struggling to promote DNA demethylation. Another possibility is that slow blastocysts present a higher number of defective mitochondria, thus an increase in TCA cycle activity could represent an attempt to produce enough energy to sustain development, but at the cost of neglecting to promote the correct DNA demethylation program. Although gene expression is not necessarily correlated with enzyme activity, this analysis allowed the exploratory evaluation of transcripts related to energy metabolism and DNA methylation reprogramming simultaneously. 

Corroborating our speculations, the increase in α-KG and succinate quantity has been already reported to promote histone and DNA demethylation, maintaining the pluripotency of embryonic stem cells [2]. Although further studies are required, it is plausible that the aberrant DNA methylation reprogramming and the metabolic disturbance (in terms of controlling energy metabolism) observed in slow embryos during development may lead to implantation failure and lower chances of offspring survival, as already reported in cloned embryos (reviewed by [28]).

In the second part of this study, we aimed to determine if the TCA cycle activity could, in fact, be responsible for epigenetic differences in bovine embryos. Therefore, we supplemented embryo culture media with different doses of α-Ketoglutarate or succinate and assessed the levels of 5mC. Changes in α-KG and succinate quantity were sufficient to interfere with DNA methylation/demethylation dynamics. Embryos cultured in the presence of higher levels of α-KG presented lower levels of DNA methylation, while supplementation with succinate induced the opposite effect. It is not clear though, if the modulation with TCA metabolites favors sex-specific losses.

The consequences of α-KG supplementation during in vitro culture were recently described in murine embryos [5], in which α-KG treatment affected TET activity leading to an increase in the 5hmC/5mC ratio, improving cell differentiation and embryo development. In the present study, blastocyst rates and total cell number were barely affected by the modulation of metabolites. Similar outcomes were already reported in different studies, in which modulators of metabolism, such as antioxidants and growth factors, altered the physiological response without affecting morphological parameters and blastocyst rates [29,30,31,32,33]. These results reinforce the importance of pursuing the improvement of IVP systems beyond the morphological aspect (those mostly used for the assessment of IVP success or embryo viability). It is clear that the composition of culture medium alters the molecular dynamics and is capable of imprinting marks that will be carried out throughout embryonic development, with possible consequences even in their adult life. Although the results presented in this study shed a light into the metabolic regulation of DNA methylation, further investigation is still required to understand whether this is a direct response of TET enzymes activity or if there are other related mechanisms involved, such as the hypoxic response promoted by hypoxia inducible factor (*HIF-1α*) or a histone-induced response promoted by the Jumonji C (Jmj-C) domain containing lysine demethylases [34].

Either way, to our knowledge, this is the first report of how metabolism and epigenetic reprogramming of bovine embryos are connected, bringing the concept of metaboloepigenetics to the developmental context. It is important to consider that the oocyte environment is related to the kinetics of the first cleavages and, consequently, the ability to correctly reprogram the epigenome or activate metabolic pathways, indicating a strong maternal contribution for the epigenetic profile of the embryo [35]. Thus, taken together, our results open new possibilities for further studies that consider metabolism and epigenetics as related processes. In addition, these results reinforce the idea that changes applied to in vitro systems must be made carefully as embryonic metabolism interventions might increase blastocyst rates, but also impair the proper molecular reprogramming, leading to medium- and long-term consequences to the offspring, even in their adult life and possibly to their future generation.

## 4. Materials and Methods

### 4.1. Chemicals and Reagents

Tissue culture media (TCM) 199-Hepes, sodium bicarbonate and fetal calf serum (FCS) were obtained from Thermo Fisher Scientific (Whaltham, MA, USA). All the other chemicals were obtained from Sigma-Aldrich (St. Louis, MO, USA), unless otherwise specified.

### 4.2. Experimental Design

The experimental design is illustrated in Supplementary Figure S2. In detail, for experiment 1, bovine embryos were produced in vitro and classified as fast (F) or slow (S) at 40 h post-insemination (hpi). Then, embryos were collected for 5mC and 5hmC immunostaining at 3 time points: 40 hpi (first cleavages—CL; fast—FCL; slow—SCL), 96 hpi (genome activation—GA; fast—FGA; slow—SGA), and 186 hpi (blastocyst—BL; fast—FBL; slow—SBL). Additionally, we analyzed the transcriptional profile of genes coding for enzymes related to energy metabolism and DNA methylation. RNA-Seq data for FBL and SBL were obtained from a previous study of the group and gene expression was validated for this study by quantitative polymerase chain reaction (PCR) [10]. In experiment 2, bovine embryos were produced in vitro and randomly assigned to the study groups at 8 hpi. Study groups were defined according to the following culture media supplementations: control medium (SOFaa + 4% BSA), α-KG (1, 2 or 4 mM in control medium) and succinate (1, 2 or 4 mM in control medium). Embryos were cultured in their respective supplementation until day 4, then transferred to new drops of culture media without α-KG or succinate supplementation where they remained until day 7. Finally, blastocysts collected at day 7 were immunostained for 5mC and total cell number (Supplementary Figure S2). In experiment 1, we evaluated a total of 52 slow embryos and 61 fast embryos for 5mC or 5hmC. RNA-seq data were previously produced using pools of 10 embryos. In experiment 2, for α-KG supplementation, we analyzed a total of 27 embryos and 30 embryos for succinate supplementation.

### 4.3. In Vitro Embryo Production (IVP)

In vitro maturation, fertilization and culture were performed as previously described [10]. For experiment 1, after 22 h from the beginning of IVC (40 hpi), embryos were classified as slow (2–3 cells) or fast (4 or more cells) and transferred separately to 90 μL droplets of synthetic oviduct fluid (SOFaa) supplemented with 2% essential amino acids, 1% nonessential amino acids and 5% FCS. Embryos were collected at 40 hpi (fast—FCL; slow—SCL), 96 hpi (fast—FGA; slow—SGA) and 186 hpi (fast—FBL; slow—SBL).

In experiment 2 (modulation of α-KG and succinate in culture media), at 8 hpi, presumptive zygotes were transferred to 90 μL droplets of semi-defined control medium (SOFaa supplemented with 4 mg/mL of BSA and essential and nonessential amino acids) or to control medium with the addition of 1, 2 or 4 mM of dimethyl-α-ketoglutarate (α-KG—349631) or 1, 2 or 4 mM of dimethyl-succinate (SUC—W239607). At day 4 [2], embryos from all groups were transferred to 90 μL droplets of control culture medium and collected at day 7 for analysis. For both experiments, embryos were cultured at 38.5 °C, 5% CO_2_ in air and high humidity.

### 4.4. Immunofluorescence for 5mC and 5hmC

For immunofluorescence of 5mC and 5hmC, embryos were fixed in 4% paraformaldehyde (*w*/*v* in phosphate buffered saline; PBS) for 15 min at room temperature (RT), washed in PBS with 1% of polyvinylpyrrolidone (PVP), permeabilized for 15 min with 0.2% TritonX-100 in PBS solution at RT, washed in PBS/PVP 1%, denatured with acid solution (3 N HCl in water) for 30 min and neutralized in basic solution (100 mM Tris-base in water) for 10 min at RT. Subsequently, embryos were washed in PBS/PVP 1% and blocked with blocking solution (0.05% of TritonX-100 and 2% of BSA diluted in PBS with 1% of PVP) over-night at 4 °C. Incubation with primary antibodies (5mC—39649 Active Motif Carlsbad, CA, USA; 5hmC— ab214728 Abcam Cambridge, UK) was done for 2 h at RT and washed in blocking solution. Next, embryos were incubated with secondary antibodies (Donkey anti-mouse— ab150109 Abcam Cambridge, UK; Donkey anti-rabbit— A21206 Life Technologies, Carlsbad, CA, USA) for 1 h at RT and then washed again in blocking solution. All antibodies were diluted in blocking solution at the moment of use. Finally, embryos were then stained with Propidium Iodate (0.05 mg/mL) for nuclear identification and total cell count, placed in slides and analyzed under a fluorescence microscope (Leica Microsystems, Wetzlar, HE, Germany, DM16000 B coupled with fluorescence filters Y3 [red—excitation/emission 538–617 nm] and L5 [green—excitation/emission 512–542 nm]). Images were acquired by the camera coupled to the microscope (Leica DFC365 FX—Leica Microsystems, Wetzlar, HE, Germany) and processed by LAS X Life Science Software (Leica Microsystems, Wetzlar, HE, Germany) with the same parameters.

### 4.5. RNA-Seq Analysis

To better understand the differences regarding the epigenetic reprogramming of fast and slow embryos, data from a previous RNA-Seq study [10] were assessed. The study investigated the transcription profile in blastocysts produced and classified with the same kinetics model described in the present work. RNA-Seq was performed using pools of 10 blastocysts per group in 3 replicates. RNA isolation, amplification and cDNA synthesis were performed as described elsewhere [10]. Briefly, amplified cDNA was quantified, and the correct size distribution was produced using Bioanalyzer (Agilent Technologies, Inc., Santa Clara, CA, USA). cDNA was amplified, fragmented and used for the construction of indexed libraries. Quality parameters were checked, and samples sequenced as 100-bp single-end reads at the UC Berkeley Vincent J. Coates facility using an Illumina HiSeq 2000 (Illumina, Inc., San Diego, CA, USA). Reads were trimmed, assessed for quality control and aligned to the UMD 3.1.71 annotated assembly using the CLC Genomics Workbench 4.7 software (CLC bio, Aarhus, Denmark). Further details explaining the complete processes are available in Milazzotto et al., 2016 [10]. Each transcript was selected based on previous reports of their role on the establishment of epigenetic marks (DNA methylation) or related metabolism, such as those from the one-carbon cycle (1C), glycolysis and the TCA cycle [36] (Supplementary Table S1). Gene counts data from transcripts of interest were subjected to DESeq2 analysis using R (3.1.0) [37] to verify differences in gene expression between groups.

### 4.6. RNA-Seq Validation

Seventeen genes were selected from pathways of interest (Supplementary Table S2) and analyzed by real-time PCR using the BioMark™ HD platform (Fluidigm, San Francisco, CA, USA) and the methodology previously described [38]. Briefly, total RNA of three blastocysts per replicate per group (four biological replicates per group: FBL and SBL) was extracted with PicoPure^®^ RNA Isolation Kit (Applied Biosystems, Waltham, MA, USA), adding the DNAse treatment, as described by the manufacturer. cDNA synthesis was performed from 10 µL of total RNA with high-capacity cDNA reverse transcription kit (#4368814, Applied Biosystems, Waltham, MA, USA) following the manufacturer’s recommendation.

cDNA was pre-amplified for 14 cycles using PreAmp Master Mix (Applied Biosystems, Waltham, MA, USA) and containing all TaqMan assays (Applied Biosystems, Waltham, MA, USA) following the manufacturer’s recommendation. Pre-amplified products were diluted 6-fold prior to RT-qPCR analysis. Chip preparation was performed as described by the manufacturer (Fluidigm, San Francisco, CA, USA) as follows: Assay Solution containing TaqMan assay and Assay Loading Reagent (Fluidigm, San Francisco, CA, USA) were allocated at the assay inlets. Sample Solution containing TaqMan Master Mix (Applied Biosystems, Waltham, MA, USA), Sample Loading Reagent (Fluidigm, San Francisco, CA, USA) and pre-amplified cDNA were allocated at the sample inlets. The chip (96.96 Dynamic Array^TM^ IFC) was transferred to the BioMark™ HD system (Fluidigm, San Francisco, CA, USA) for RT-qPCR according to the TaqMan GE 96 × 96 Standard protocol.

### 4.7. Statistical Analysis

Immunofluorescence images were analyzed by the ImageJ 1.52q software (U. S. National Institutes of Health, Bethesda, MD, USA). The mean fluorescence intensity of each nuclei area was quantified with background subtraction. For each modification (5hmC and 5mC), data were obtained from at least 4 biological replicates. The number of nuclei assessed for each group varies between 17 and 54 for CL, 69 and 163 for GA and 310 and 576 for the BL groups in experiment 1. In experiment 2, data were obtained from 446–582 nuclei at day 7. Only nuclei with the DNA clearly visible by Propidium Iodate were analyzed. The distinction between ICM and TE cells was performed visually according to morphological parameters described elsewhere [39]. Blastocyst rates were calculated using the number of cleaved embryos over the total number of blastocysts per group. Total cell number of each blastocyst was counted using the ImageJ 1.52q software.

All obtained values were submitted to an outlier detection test (Grubbs’ test with alpha = 0.05) and the normality test of D’Agostino and Pearson. In case of normal distribution, a Student’s *t*-test was performed for comparisons between fast and slow groups and ANOVA followed by the Dunn test for comparison among developmental stages in the two experimental designs (fast vs. slow and α-KG/succinate quantity modulation). In case of non-parametric data, the Mann–Whitney test was performed for kinetics comparison (fast vs. slow) and Kruskal–Wallis for developmental stage comparisons. Results are presented in graphs for each epigenetic mark and the fluorescence intensity is represented as arbitrary units (A.U.). For experiment 1, fluorescence intensities of 5mC and 5hmC were normalized (by scaling from 0 to 1) and their means were used to calculate a ratio (5mC/5hmC), as described by [40]. The results are presented as pie charts and represent the overall change in proportion between the two marks.

For RNA-Seq analysis, data from fast and slow blastocysts were submitted to principal component analysis (PCA; ClustVis [41]) and gene ontology (David: Functional Annotation Bioinformatics Microarray Analysis [42,43]) to evidence the most affected biological processes. Gene counts for each transcript of interest were analyzed using DESeq2 with R 3.6.3 [37]. The complete list of selected genes is detailed in Supplementary Table S1. Genes presenting *p*-Adj > 0.1 were considered as not statistically different, *p*-Adj ≤ 0.1 and > 0.05 were considered as different and *p*-Adj ≤ 0.05 as with a strong difference.

To validate RNA-Seq data, 17 target genes were analyzed by RT-qPCR using the BioMark™ HD system (Fluidigm, San Francisco, CA, USA). The Ct value for each target gene was normalized by *PPIA* selected from three reference genes with NormFinder (Department of Molecular Medicine, Aarhus University Hospital, Aarhus, Dinamarca) [44]. ΔCt values were calculated, tested for the presence of outliers and compared to RNA-Seq data to assess whether they were consistent using a Student’s *t*-test by the Prism 5 software (GraphPad Software Inc., San Diego, CA, USA). Genes presenting *p* ≤ 0.05 were considered as statistically different. Data from gene expression and their relationship with RNA-Seq are described in Supplementary Table S2. The figures were prepared using the models provided by Servier Medical Art (https://smart.servier.com).

## Supplementary Materials

The following are available online at https://www.mdpi.com/1422-0067/21/18/6868/s1, Figure S1: (A) Total number of cells in fast (FBL) and slow (SBL) blastocysts. (B) Fluorescence intensity for 5-methylcytosine and 5-hydroxymethylcytosine of fast and slow blastocysts of cells from Trophoectoderm (TE) or inner cell mass (ICM). Fluorescence intensity for 5-methylcytosine of cells from the ICM or TE in blastocysts cultured with (C) dimethyl-succinate or (D) dimethyl-α-ketoglutarate. Statistical significance is identified by different letters, Figure S2: Experimental design, Table S1: Selected genes related to metabolism and epigenetic mechanisms from RNA-Seq analysis of bovine blastocysts (slow vs. fast). Genes in blue represent upregulation in slow blastocysts, genes in red represent upregulation in fast blastocysts, Table S2: Validation of RNA-Seq results by correlation with RT-qPCR analysis of genes related to epigenetic mechanisms, mitochondrial apoptosis and embryonic pluripotency.
