# Supplementary material for: Tricarboxylic Acid Cycle Metabolites as Mediators of DNA Methylation Reprogramming in Bovine Preimplantation Embryos
Source: Int J Mol Sci. 2020 Sep 18;21(18):6868. doi: 10.3390/ijms21186868 (PMC7558971; doi:10.3390/ijms21186868)
Supplement: Supplementary file 1 [file ijms-21-06868-s001.pdf]

Supplementary Materials

Tricarboxylic Acid Cycle Metabolites as Mediators of DNA Methylation Reprogramming in Bovine Preimplantation Embryos

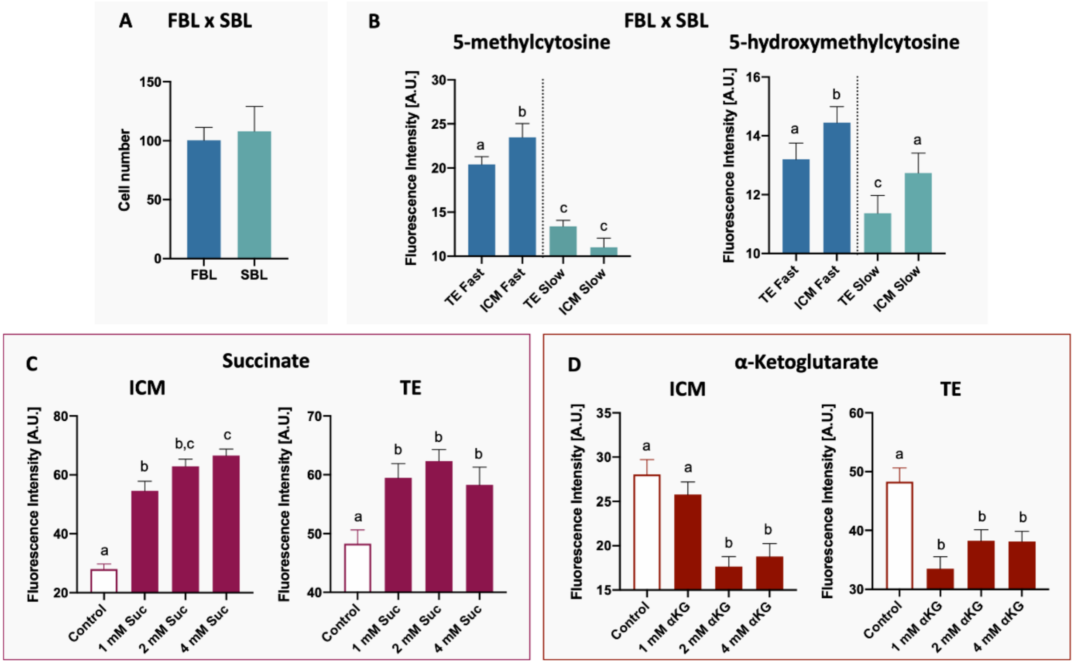

Figure S1. (**A**) Total number of cells in fast (FBL) and slow (SBL) blastocysts; (**B**) Fluorescence intensity for 5-methylcytosine and 5-hydroxymethylcytosine of fast and slow blastocysts of cells from Trophoectoderm (TE) or inner cell mass (ICM). Fluorescence intensity for 5-methylcytosine of cells from the ICM or TE in blastocysts cultured with (**C**) dimethyl-succinate or (**D**) dimethyl-*α*-ketoglutarate. Statistical significance is identified by different letters.

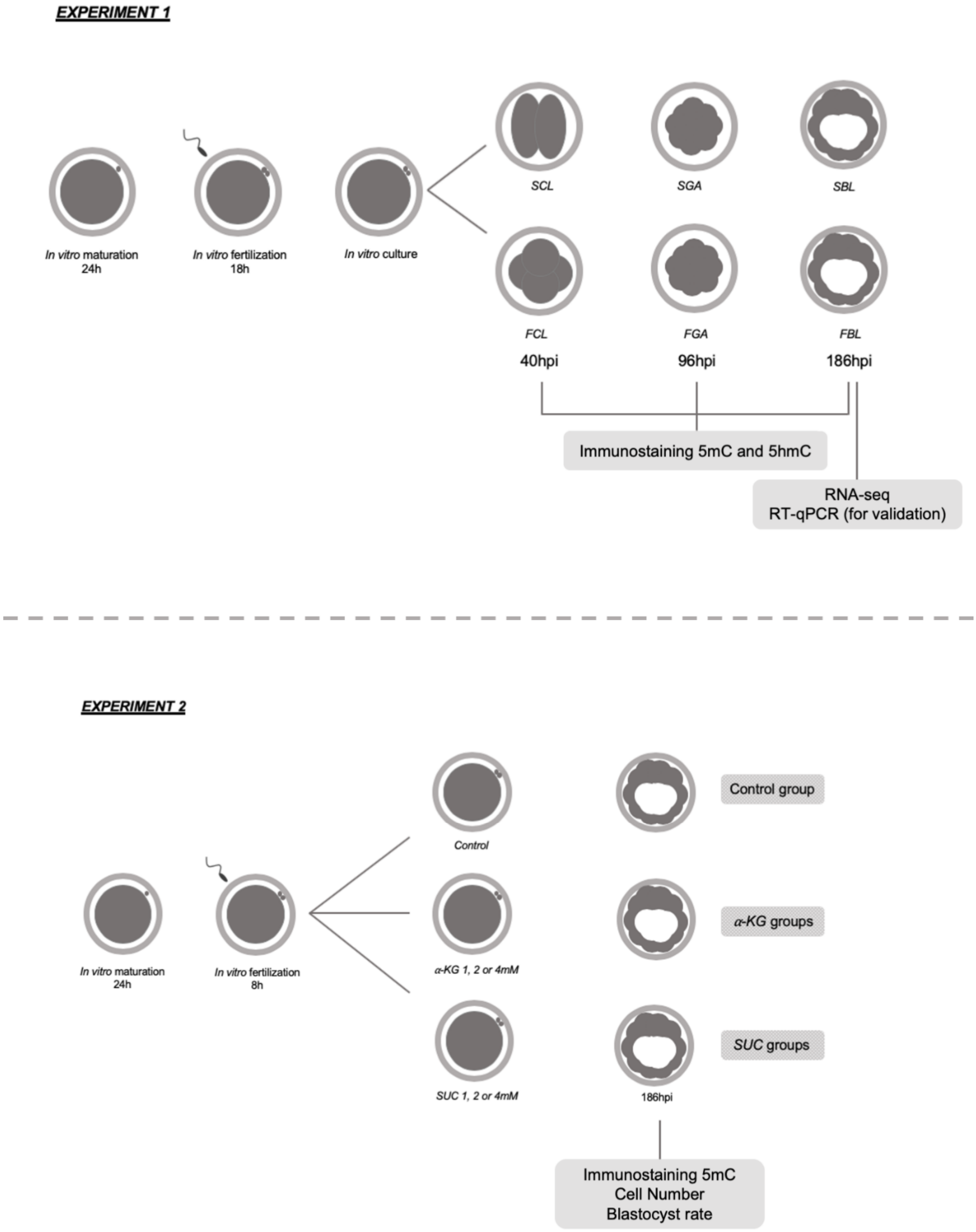

Figure S2. Experimental design.

Table S1. Selected genes related to metabolism and epigenetic mechanisms from RNA-Seq analysis of bovine blastocysts (slow *vs.* fast). Genes in blue represent upregulation in slow blastocysts, genes in red represent upregulation in fast blastocysts.

Gene	log2FoldChange	*p*-value	*p*-Adj
PDHB	−1.425	0.000	0.000
MDH1	−1.206	0.000	0.000
APEX1	−1.193	0.000	0.000
OGDHL	−3.417	0.000	0.002
PGK1	−0.942	0.000	0.002
GLS2	1.493	0.000	0.002
AICDA	1.171	0.001	0.005
ACO2	0.693	0.002	0.011
CS	−0.660	0.002	0.011
SLC25A1	1.181	0.007	0.032
IDH3A	−0.728	0.008	0.035
GSS	1.039	0.013	0.053
TET3	0.662	0.026	0.093
GLUD1	−0.450	0.032	0.108
SDHD	−0.619	0.049	0.143
FH	−0.547	0.054	0.149
OGDH	0.316	0.133	0.287
ACO1	−0.364	0.141	0.297
SDHC	−0.335	0.149	0.311
LIG3	0.338	0.165	0.334
SUCLG	−0.332	0.174	0.349
SDHA	0.297	0.210	0.396
SUCLA2	−0.324	0.248	0.439
DNMT1	0.266	0.279	0.486
IDH3B1	−0.269	0.296	0.503
SDHB	−0.213	0.339	0.544
DNMT3B	0.181	0.386	0.598
APOBEC1	0.629	0.386	0.598
TDG	0.427	0.398	0.611
IDH3G	0.237	0.468	0.675
NEIL2	0.509	0.572	0.720
IDH2	0.298	0.571	0.720
DNMT3L	1.306	0.590	0.722
GLS	0.120	0.706	0.821
XRCC1	0.108	0.793	0.887
TET1	−0.028	0.879	0.919
DNMT3A	0.029	0.893	0.920
MBD4	−0.056	0.885	0.920
PDHX	0.033	0.890	0.920
SMUG1	0.053	0.936	0.954
TET2	−0.002	0.991	0.991

Table S2. Validation of RNA-Seq results by correlation with RT-qPCR analysis of genes related to epigenetic mechanisms, mitochondrial apoptosis, and embryonic pluripotency.

Pathway	Gene	Gene ID	TaqMan Code	Uniprot Entry	RT-qPCR FBL	RT-qPCR SBL	RT-qPCR *P* value	RNA-Seq FBL	RNA-Seq SBL	RNA-Seq *P* value	Relation
Epigenetic	DNA methyl-transferase 1	DNMT1	Bt03224737_m1	BT.108052	0.21 ± 0.02	0.20 ± 0.02	0.3693	10.46 ± 0.18	10.17 ± 0.13	0.258	Equal
	DNA methyl-transferase 3A	DNMT3A	Bt01027164_m1	BT.64560	1.21 ± 0.26	0.86 ± 0.11	0.1438	13.26 ± 0.09	13.19 ± 0.20	0.806	Equal
	DNA methyl-transferase 3B	DNMT3B	Bt03259810_m1	BT.22977	0.41 ± 0.04	0.50 ± 0.04	0.1032	12.87 ± 0.02	12.65 ± 0.18	0.321	Equal
	H1 histone member O	H1FOO	Bt03228652_g1	Bt.36838	0.14 ± 0.02	0.12 ± 0.00	0.2472	6.48 ± 0.68	6.85 ± 1.06	0.329	Equal
	H3 Histone, Family 3A	H3F3A	Bt03278804_g1	Bt. 60099	0.21 ± 0.01	0.26 ± 0.01	0.0149	11.82 ± 0.13	13.28 ± 0.28	3.03^{-03}	Equal
	H3 Histone, Family 3B	H3F3B	Bt04319377_g1	Bt.15474	0.27 ± 0.01	0.26 ± 0.01	0.1404	4.79 ± 0.42	5.22 ± 0.87	0.471	Equal
	Heterochromatin protein 1	HP1	Bt03246076_m1	BT.22333	0.20 ± 0.01	0.21 ± 0.00	0.2995	12.20 ± 0.14	12.02 ± 0.12	0.401	Equal
	H2A.Z Variant Histone	H2AFZ	Bt03216346_g1	Bt.2515	1.59 ± 0.33	1.32 ± 0.21	0.2700	11.29 ± 0.26	12.83 ± 0.40	3.44^{-06}	Different
	Histone Deacetylase 2	HDAC2	Bt03244871_m1	Bt.27729	0.23 ± 0.00	0.23 ± 0.00	0.4848	10.08 ± 0.21	10.57 ± 0.13	0.065	Different
Mitochondrial apoptosis	Caspase 3	CASP3	Bt03250954_g1	Bt.10084	0.14 ± 0.01	0.15 ± 0.01	0.3283	9.45 ± 0.61	9.49 ± 0.17	0.802	Equal
	Caspase 9	CASP9	Bt04282453_m1	Bt.66332	0.19±0.00	0.18±0.00	0.0088	8.97 ± 0.22	7.90 ± 0.10	0.003	Equal
	BCL2 Associated X, Apoptosis Regulator	BAX	Bt03211777_g1	Bt.109788	0.66 ± 0.01	0.56 ± 0.04	0.0272	8.68 ± 0.41	8.25 ± 0.35	0.273	Different
	BH3 Interacting Domain Death Agonist	BID	Bt03241255_m1	Bt.87470	0.15 ± 0.00	0.17 ± 0.01	0.0348	9.79 ± 0.17	8.96 ± 0.41	0.022	Opposite
Embryonic pluripotency	Nanog Homeobox	NANOG	Bt03220541_m1	Bt.47449	0.15 ± 0.00	0.19 ± 0.00	0.0016	8.84 ± 0.35	9.64 ± 0.06	0.031	Equal
	POU Class 5 Homeobox 1	POU5F1	Bt03223846_g1	BT.92603	0.85 ± 0.08	0.57 ± 0.06	0.0173	11.87 ± 0.26	11.66 ± 0.28	0.444	Different
	SRY-Box Transcription Factor 2	SOX2	Bt03278318_s1	BT.103364	0.25 ± 0.03	0.25 ± 0.00	0.4821	8.11 ± 0.19	7.31 ± 0.60	0.195	Equal
	Caudal Type Homeobox 2	CDX2	Bt03649157_m1	Bt.46244	0.33 ± 0.01	0.27 ± 0.01	0.0100	10.30 ± 0.38	9.07 ± 0.48	0.001	Equal

(1) The genes *PPIA* (Bt03224617_g1; Bt.43626), *ACTB* (PA5-16914; BT.14186), and *GAPDH* (Bt03210912_g1; Bt.87389) were also analyzed as reference genes and submitted to Normfinder evaluation. PPIA was used for data normalization; (2) From the 17 genes analyzed, 70.58% (12) presented the same pattern in both experiments, while 23.5% (4) were statistically different only in one of the analysis. Only one gene (5.9%) had the opposite pattern between the two techniques.
